# Efficacy of acupuncture for chronic prostatitis/chronic pelvic pain syndromes: study protocol for a randomized, sham acupuncture-controlled trial

**DOI:** 10.1186/s12906-016-1428-y

**Published:** 2016-11-07

**Authors:** Zongshi Qin, Zhiwei Zang, Jiani Wu, Jing Zhou, Zhishun Liu

**Affiliations:** 1 Department of Acupuncture, Guang’anmen Hospital, China Academy of Chinese Medical Sciences, Beijing, 100053 China; 2 Beijing University of Chinese Medicine, Beijing, 100029 China; 3 Department of Acupuncture, Yantai Hospital of Traditional Chinese Medicine, Yantai, 265200 China

**Keywords:** Chronic prostatitis/chronic pelvic pain syndrome, Acupuncture, Efficacy, Randomized controlled trial

## Abstract

**Background:**

Chronic prostatitis/chronic pelvic pain syndrome (CP/CPPS) affects many adult men worldwide. The currently available therapies offer little or no proven benefit for CP/CPPS. We designed this study to assess the efficacy of acupuncture therapy for the treatment of CP/CPPS.

**Methods:**

This study is designed as a randomized, sham acupuncture-controlled trial. We will compare patients with CP/CPPS in an acupuncture group and a sham acupuncture group. Sixty-eight patients will be randomly allocated to receive acupuncture or sham acupuncture. The treatments will consist of 30-min sessions, three times weekly, for 8 weeks. The primary outcome measure is change in the weekly mean National Institutes of Health Chronic Prostatitis Symptom Index (NIH-CPSI) total score from baseline through the 8-week treatment period. Secondary measures include the NIH-CPSI subscale scores, the total International Prostate Symptom Score (IPSS), patients’ response rate, and patient satisfaction after treatment. We will also assess changes in the NIH-CPSI total score from baseline at the 20^th^ and 32^nd^ week of follow-up.

**Discussion:**

This is a randomized, sham-controlled trial of acupuncture treatment for CP/CPPS. The results of this trial will provide more evidence on whether acupuncture is efficacious for treating CP/CPPS.

**Trial registration:**

Clinical Trials.gov NCT02588274

## Background

Chronic prostatitis/chronic pelvic pain syndrome (CP/CPPS) is a common prostatic syndrome with a worldwide prevalence of 2 % to 10 % in adult men [1–3]. Based on a survey in China, the prevalence of CP/CPPS-like symptoms among Chinese men is 4.5 % [4]. CP/CPPS can present with a wide range of clinical manifestations; the main symptoms include urogenital pain, lower urinary tract symptoms, psychological issues and sexual dysfunction [5]. Compared with other urological conditions, the aetiologic factors of CP/CPPS are unclear, and its pathophysiological mechanisms are still poorly understood. It is hypothesized that inflammation or abnormal activity of the pelvic nerve and muscle play important roles in this disease [6]. According to the consensus guidelines, there is still no standard treatment for CP/CPPS; as a result, individualized therapy and symptom-based treatment approaches are recommended [5]. The interventions for CP/CPPS include medication (alpha-adrenergic antagonists, antibiotics, pain pharmacotherapies, 5-alpha-reductase inhibitors, and phytotherapy), physiotherapy (biofeedback, acupuncture) and surgical intervention [7–10]. Drugs such as alpha-adrenergic antagonists and antibiotics have been considered the initial treatment options for CP/CPPS, but in most cases, the administration of a single drug does not relieve multiple symptoms [5]. Hence, other approaches to prevent and ameliorate CP/CPPS symptoms are considered essential. Acupuncture may be a currently underestimated option for CP/CPPS treatment. Based on prior research by Lee et al., acupuncture may relieve the pain-related symptoms of CP/CPPS [11]. Thus far, although several well-designed randomized controlled trials (RCTs) related to acupuncture for the treatment of CP/CPPS have been published [12–15], the recommendations for CP/CPPS in current guidelines remain at level 5 (the lowest level) [5]. Therefore, we designed this trial to further assess the efficacy of acupuncture for treating CP/CPPS.

## Methods/design

### Aim

The aim of this study is to evaluate the efficacy of acupuncture in CP/CPPS patients.

### Design

**Fig. 1:**
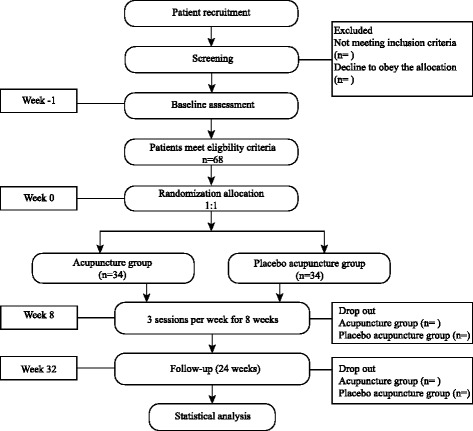
Flow chart

This study is a multi-centre, sham-controlled, randomized trial of acupuncture for the treatment of CP/CPPS that will be conducted in the Guang’anmen Hospital of China Academy of Chinese Medical Sciences and Yantai Hospital of Traditional Chinese Medicine from November 2015 to May 2017. All of the patients will be asked to sign an informed consent form prior to randomization. Blinded evaluators and statisticians will manage all data. We developed this protocol according to the Standard Protocol Items: Recommendations for Interventional Trials (SPIRIT) checklist [16]. This trial protocol has been approved by the Research Ethical Committee of Guang’anmen Hospital of China Academy of Chinese Medical Sciences and Yantai Hospital of Traditional Chinese Medicine, and it has been registered under the identifier NCT02588274 at ClinicalTrials.gov in the USA. Figure 1 provides a study flow chart.

### Participants

Sixty-eight patients will be recruited from the Guang’anmen Hospital of Chinese Academy of Chinese Medical Sciences and Yantai Hospital of Traditional Chinese Medicine.

### Inclusion criteria

Patients must meet the diagnostic criteria from the NIH CP/CPPS consensus [17], including the following:

1. History of pain perceived in the prostate region and absence of other lower urinary tract pathology for a minimum of three of the past 6 months. In addition, any associated lower urinary tract symptoms, sexual function, and psychological factors should be addressed. Physical examinations, urine analyses, and urine cultures will be performed for all subjects.
2. Age 18 to 50 years
3. NIH Chronic Prostatitis Symptom Index (NIH-CPSI) total score ≥ 15 (scale 0–43, with 0 meaning no symptoms).

### Exclusion criteria

1. Other urologic diseases, such as acute prostatitis, bacterial prostatitis, benign prostatic hyperplasia (BPH), prostate cancer, urinary tuberculosis, and urinary tract infection. (The 2-glass test will be performed in patients with urinary tract symptoms using their voided bladder 2 (VB2) and VB3, which can provide fairly accurate results and is easy to perform [18].)
2. Serious or acute diseases involving the heart, liver, kidney or blood.
3. Patients receiving acupuncture or medication (including alpha blockers or pain killers) in the week prior to the baseline assessment.

### Recruitment procedures

We will recruit the participants using advertisements in newspapers, on television, and on the Internet. Prospective participants will obtain a good understanding of the trial by reading the advertisement, which will include a brief introduction of the population needed, the contact information of the researchers, and details of the acupuncture intervention and of the comparison (all participants will be informed that there are two acupuncture groups, i.e., a traditional acupuncture group and a sham acupuncture group, in which blunt needles are used to stimulate the acupoint skin, and that they have a 50 % chance of being allocated into either of the two groups). If the patient is eligible and interested in the trial, they will be invited to consult with the study doctors; after the doctors have provided a diagnosis, the patients will be recruited. All of the participants will be required to sign an informed consent form before the trial that will include an introduction of CP/CPPS, the inclusion and exclusion criteria of the trial, and an introduction to the interventions. In addition, participants have the right to withdraw from the trial at any time.

### Randomization

After the participants have completed a baseline evaluation and met the selection criteria, one research assistant, uninvolved with the treatment and data collection, will be responsible for randomly grouping the participants. The acupuncturists will be blinded to the process of randomized assignment. The random sequence will be generated by the Institute of Clinical Pharmacology affiliated to Guang’anmen Hospital of China Academy of Chinese Medical Sciences. Randomization numbers using a block of four will be sealed in a scheduled computer-generated opaque randomization envelope. To ensure that randomization is successfully implemented, the patient’s sequence number will be written outside of the envelope, and a paper-written group name will be inside the envelope. All envelopes will be numbered sequentially. The envelopes will be delivered according to the patients’ screening sequence numbers. Finally, the acupuncturist will be informed of the random numbers and the group assignment by telephone or e-mail.

### Interventions and comparison

#### Treatment group

According to records in the ancient Traditional Chinese Medicine work *Huangdi Neijing*, acupuncture points belonging to bladder meridian (BL) have a noticeable effect on urinary disease. Furthermore, spleen meridian (SP), kidney meridian (KI), and liver meridian (LR) intersect at Sanyinjiao (SP 6), one of the most frequently used acupuncture points for urogenital disease [19, 20]. In addition, the treatment was based on the theory of neuroanatomy [10, 21, 22] and consensus among acupuncture experts in Guang’anmen Hospital. Therefore, we chose the following acupuncture points: Zhongliao (BL 33), Shenshu (BL 23), Huiyang (BL 35), and Sanyinjiao (SP 6) (Table 1). After the patients are relaxed and in a prone position, acupuncturists will use 75 % alcohol pads to sterilize the skin around the acupuncture points and then insert steel needles (Huatuo, Suzhou, China 0.3 mm*40 mm/0.3 mm*75 mm) into the acupuncture points. For bilateral Zhongliao (BL 33), the needle will be inserted approximately 50–60 mm with a 45-degree angle. For Huiyang (BL 35), the needle will be inserted approximately 50–60 mm. For Shenshu (BL 23) and Sanyinjiao (SP 6), the needles will be inserted vertically to a depth of 25–30 mm. Acupuncturists will twirl at BL23, BL35 and SP 6 to achieve and enhance the sensation of aches, heaviness or numbness in the area surrounding the inserted needle (known as de qi), and the manipulations will be performed a total of three times during 1 session (every 10 min). For bilateral BL 33, which are located in the 3^rd^ posterior sacral foramina, needles will be inserted without lifting or rotating based on the characteristics of the points. There are 24 treatment sessions after baseline that occur three times a week, and the participants will receive a 30-min treatment each session.

**Table 1:** Measurements at different time points

Measurements	baseline	1 week	2 weeks	3 weeks	4 weeks	5 weeks	6 weeks	7 weeks	8 weeks	20 weeks	32 weeks
NIH-CPSI total score	**×**	**×**	**×**	**×**	**×**	**×**	**×**	**×**	**×**	**×**	**×**
NIH-CPSI subscales score	**×**				**×**				**×**	**×**	**×**
IPSS total score	**×**				**×**				**×**	**×**	**×**
Global response assessment	**×**				**×**				**×**	**×**	**×**
Degree of expectation	**×**										
Satisfation									**×**	**×**	**×**

*NIH-CPSI* The National Institutes of Health Chronic Prostatitis Symptom Index, *IPSS* International Prostate Symptom Score

#### Control group

**Fig. 2:**
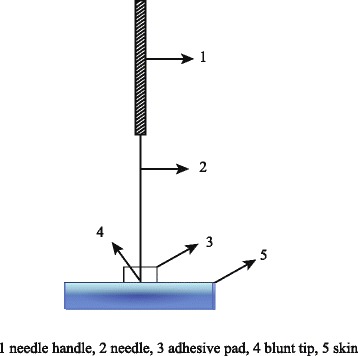
Sham acupuncture used in this trial

The control group will receive sham acupuncture at the same acupuncture points as the treatment group. The sham needle with a blunt tip used in the control group is similar to the Streitberger needle (Fig. 2 provides a diagram to illustrate the sham acupuncture applied in this trial) [23]. Acupuncturists will gently lift, thrust, and twist the sham needles to simulate the treatment procedure, thus blinding the patients to the intervention. Each acupuncture point will undergo the same twirling motion as the acupuncture group. The duration and frequency of the sessions will be the same as in the acupuncture group. To ensure blinding, the investigators will make appointments with each participant on alternate days to prevent crosstalk between groups. To test the success of the blinding, the participants will be asked to answer the following question after the 4^th^ week treatment: “Do you think you received traditional acupuncture (A) or sham acupuncture (blunt needles used to stimulate the acupoint skin) (B)?” The participants can answer “A”, “B” or “unclear” (these two interventions will be demonstrated before the treatment, and all participants will have a good understanding of both interventions). Any medication usage should be recorded, including the name of the medicine, the dosage and the session. We will compare the proportion of subjects using medicine and the mean days of medication use between the groups.

### Outcome measures

#### Primary outcome measures

The primary outcome of this study is the change in the NIH-CPSI total score, which will be measured from baseline to the 8^th^ week. After we have collected data for 8 weeks, the average score from each week will be calculated.

The NIH-CPSI is a validated, self-reported questionnaire that is widely used to assess CP/CPPS patients [24, 25]. It contains 13 items that are scored in three discrete domains: pain, urinary symptoms and impact on quality of life. A score of 0 indicates that the condition described in the question never occurs.

The secondary outcome measures include the following (for the first three secondary outcomes, we will measure the change from baseline):

1. NIH-CPSI total score at the 20^th^ and 32^nd^ week.
2. NIH-CPSI subscale scores at the 4^th^, 8^th^, 20^th^, and 32^nd^ week.
3. IPSS total score at the 4^th^, 8^th^, 20^th^, and 32^nd^ week. The International Prostate Symptom Score (IPSS) is another valid, reliable and sensitive measure for patients with lower urinary tract symptoms (LUTS). The IPSS has been translated into several languages, including Chinese, and it is widely used in clinical practice and research to determine the severity of LUTS, including incomplete bladder emptying, frequency of urination, intermittency, urgency, weak urine stream, straining and nocturia [26, 27]. Each of the questions is rated from 0 (not at all) to 5 (almost always), and according to the total symptom score, the severity of LUTS can be graded as mild (0–7), moderate (8–19) or severe (20–35).
4. Global response assessment at the 4^th^, 8^th^, 20^th^, and 32^nd^ week. Patients who have at least a 50 % decrease compared to baseline in total NIH-CPSI score will be considered “responders”. We will compare the response rate between the two groups.
5. Expectations that acupuncture might help CP/CPPS at baseline. This scale includes four brief questions to investigate whether patients are confident that acupuncture treatment will help their CP/CPPS.
6. The degree of satisfaction for patients undergoing acupuncture treatment will be measured at the 8^th^, 20^th^, and 32^nd^ week.

Table 1 shows the time to visit and the data collection measurements at different time points.

### Safety assessment

Adverse events (AEs) related to acupuncture treatment will be appropriately assessed and recorded by the observers throughout the trial, for instance, pain, haematomas, or nausea. AEs will be managed by acupuncturists and related clinical specialists within 24 h. The principal investigator (Z. Liu) will make the final decision to terminate the trial if severe AEs arise.

### Data collection and quality control

Because nonstandard input of clinical data can contribute to the bias of results, two investigators (to assess the effect of treatment) will independently collect the data using case report forms and then input the data into a computer; this process will ensure the safety and reliability of the data. To confirm the quality of the trial, all acupuncturists will be required to have an official license and more than two years of clinical experience.

### Sample size calculation and statistical analysis

The calculation of sample size was based on the primary outcome of change in NIH-CPSI total score from baseline to the 8^th^ week of treatment (mean of 8 weeks of data). According to previous literature [12], after 10 weeks of sham acupuncture treatment, NIH-CPSI scores decreased by 6.2, with a standard deviation of 10.3. We estimated that for a 13.3-point difference in the NIH-CPSI score with a standard deviation of 4.5, and based on our clinical experience and data from previous research, the decrease in the NIH-CPSI total score should be more than 6 points (i.e., the minimal clinically important difference, MCID) [28]. Therefore, we will need to recruit 34 patients per group from Guang’anmen Hospital of Chinese Academy of Chinese Medical Sciences and Yantai Hospital of Traditional Chinese Medicine to obtain 90 % power and a significance level of 5 % while allowing for a 20 % dropout rate.

The data will be analysed using SPSS software Ver.19.0 (IBM SPSS Statistics, IBM Corp, Somers, New York), and the data analysis will be based on the intention-to-treat (ITT) principle regarding baseline characteristics. If there are significantly more dropouts in the control group than in the treatment group, we will conduct a secondary analysis of subjects who have completed the treatment according to the protocol. The outcomes for evaluating changes between the groups will be based on the outcome of the NIH-CPSI at different time points. The significance levels will be two-sided and reported at a 5 % level. For continuous measurement data, the mean, standard deviation, median and interquartile range will be represented. If the data are normally distributed, analysis of covariance will be used because the centres and baseline variables in this study are covariates that are not manipulated by the researchers but may still have an effect on the efficacy of acupuncture. If the distribution is not normal, we will use generalized estimating equations, if needed, to account for the cumulative acupuncture frequency and to observe whether increasing treatment sessions results in increased efficacy for patients. Linear regression will be used to explore whether the patients’ expectations have an impact on treatment. For categorical data, a CMH (Cochran-Mantel-Haenszel) test will be used, and the data will be represented as case and percentages. Missing data will be assumed to be missing randomly and will be imputed using multiple imputation.

Table 2 summarizes the details of the analysis methods for the primary and secondary outcomes.

**Table 2:** Analysis methods for outcomes

Outcomes	Time frame	Statistics
Primary outcome		
NIH-CPSI total score and change from baseline	baseline, week 1-8	Covariance analysis or generalized estimating equations
Secondary outcomes		
NIH-CPSI subscales score	Baseline, week 4,8,20,32	Covariance analysis or generalized estimating equations
NIH-CPSI total score in follow-up	week 20,32	Covariance analysis or generalized estimating equations
IPSS total score and change from baseline	baseline, week 4,8,20,32	Covariance analysis or generalized estimating equations
Global response assessment improvement	week 4,8,20,32	CMH test or nonparametric test
Degree of expectation	baseline	linear regress
Degree of satisfaction	week 8,20,32	CMH test or nonparametric test

## Discussion

Acupuncture has been used to treat urinary diseases in China for centuries, and many RCTs have been focused on this illness [29–33]. Several RCTs to date have shown that acupuncture or electro-acupuncture is effective for CP/CPPS [12–14]; however, according to a systematic review and the consensus guidelines, positive evidence for acupuncture-based therapy in CP/CPPS remains poor [17, 34]. The lack of evidence is mainly attributable to the methodological limitations of prior studies, such as inappropriate trial designs, inadequate control groups, lack of follow-up, and lack of suitable outcome measures. Some well-designed trials have indicated that the effect of invasive sham acupuncture may be comparable to that of real acupuncture or standard drug therapy [35, 36]. These results might call into question whether the “invasive sham acupuncture” is a reasonable control for CP/CPPS or whether it is actually a type of shallow acupuncture intervention. Several researchers have also argued about whether invasive sham procedures can be incorporated by acupuncture trials [37, 38]. We are also curious about this question, and we designed the sham acupuncture to use a blunt-tip needle on acupoints without penetration. According to the findings of our prior crossover study, this non-insertion-type needle is a valid sham control for acupuncture research and can achieve good subject blinding effects with a similar appearance to traditional acupuncture [39]. Given a sufficiently thin needle and the manipulation used (lifting, thrusting or rotating), even blunt tips can make participants feel the sensation of piercing. Most of the participants were hardly able to distinguish this device from real acupuncture in the previous trial [39]. In addition, in the current trial, we will use BL 23, BL 33, BL 35 and SP 6 as the selected acupoints instead of conception vessel 1 (CV 1) and CV 4. According to our previous clinical experiences and studies related to BPH, acupuncture on BL 23, BL 33, and BL 35 could significantly improve the condition of patients with LUTS [40]. Furthermore, the selected acupoints mentioned above are located on the participant’s lower back and distal lower extremity; therefore, the participants will barely be able to see the treatment procedure and puncture wounds from their prone position. Thus, we anticipate that the acupoints chosen in this trial might increase the possibility of patient-blinding.

In this trial, we aim to clarify the efficacy and safety of acupuncture for treating CP/CPPS. We will calculate the mean NIH-CPSI scores over 8 weeks, and these scores will indicate the weekly efficacy of acupuncture during the 8 weeks of treatment; additionally, the mean value may provide more sufficient evidence for determining whether acupuncture is efficacious for treating CPPS. Furthermore, we will add an expectations scale to explore whether the patients’ expectations have a potential impact on the outcomes of acupuncture treatment. Nevertheless, this study has several limitations, including the small sample size and the unblinded acupuncturists. Due to the use of a sham acupuncture control, a relatively high rate of dropouts might be observed in the control group. Additionally, there is a risk that the blinding method may be unsuccessful in control participants, as the blunt tip needle will not leave puncture holes. Finally, a few participants might be recruited mistakenly due to the overlapping symptoms of CP/CPPS and BPH [41]. Nonetheless, the outcome assessors and patients will be blinded to decrease the potential for bias [42]. We hope that the results of this trial can provide both an evidence-based treatment option for patients suffering from CP/CPPS and an enhanced level of evidence on which to base guideline recommendations.

## Abbreviations

AEs: Adverse events
BL: Bladder meridian
BPH: Benign prostatic hyperplasia
CMH: Cochran-Mantel-Haenszel
CP/CPPS: Chronic prostatitis/chronic pelvic pain syndrome
CV: Conception vessel
IPSS: International Prostate Symptom Score
ITT: Intention-to-treat
KI: Kidney meridian
LR: Liver meridian
LUTS: Lower urinary tract symptoms
NIH-CPSI: National Institutes of Health Chronic Prostatitis Symptom Index
RCTs: Randomized controlled trials
SP: Spleen meridian
SPIRIT: Standard Protocol Items: Recommendations for Interventional Trials
VB: Voided bladder
